# The Structure and Function of the Na,K-ATPase Isoforms in Health and Disease

**DOI:** 10.3389/fphys.2017.00371

**Published:** 2017-06-06

**Authors:** Michael V. Clausen, Florian Hilbers, Hanne Poulsen

**Affiliations:** Department of Molecular Biology and Genetics, Aarhus UniversityAarhus, Denmark

**Keywords:** Na, K-ATPase, structure, expression, isoforms, subunits, disease

## Abstract

The sodium and potassium gradients across the plasma membrane are used by animal cells for numerous processes, and the range of demands requires that the responsible ion pump, the Na,K-ATPase, can be fine-tuned to the different cellular needs. Therefore, several isoforms are expressed of each of the three subunits that make a Na,K-ATPase, the alpha, beta and FXYD subunits. This review summarizes the various roles and expression patterns of the Na,K-ATPase subunit isoforms and maps the sequence variations to compare the differences structurally. Mutations in the Na,K-ATPase genes encoding alpha subunit isoforms have severe physiological consequences, causing very distinct, often neurological diseases. The differences in the pathophysiological effects of mutations further underline how the kinetic parameters, regulation and proteomic interactions of the Na,K-ATPase isoforms are optimized for the individual cellular needs.

## Introduction

During the evolution of life, most living cells have maintained a similar ionic composition of their cytoplasms, including low calcium, low sodium, high potassium, and neutral pH (Mulkidjanian et al., 2012). When the extracellular ionic concentrations are significantly different, it requires perpetual ion pump activity to uphold the intracellular concentrations, which are important for numerous of the cell's enzymatic functions. Furthermore, much energy is stored in the ionic gradients across the plasma membrane, and the steep sodium and potassium gradients in animal cells are used to facilitate secondary transport of molecules (sugars, neurotransmitters, amino acids, metabolites) and other ions (H^+^, Ca^2+^, Cl^−^). The ion gradients are also used for rapid signaling by opening of sodium or potassium selective channels in the plasma membrane in response to extracellular signals or the membrane potential.

Many organs use the sodium and potassium gradients for their specialized functions. In the kidneys, the Na,K-ATPase is highly expressed, an estimate says up to 50 million pumps per cell in the distal convoluted tubule (El Mernissi and Doucet, 1984), because the sodium gradient is utilized by the main kidney functions, to filter the blood of waste products, to reabsorb glucose and amino acids, to regulate electrolytes and to maintain pH. In sperm cells, the regulation of ions and membrane potential is crucial for motility and the acrosome reaction, and sperm cells express a unique Na,K-ATPase isoform, which is essential for male fertility (Jimenez et al., 2011a). Not least the brain has a massive demand for Na,K-ATPase activity, since neurons rely on the pump to reverse postsynaptic sodium flux, to reestablish the sodium and potassium gradients used to fire action potentials, and in astrocytes, the sodium gradient drives neurotransmitter reuptake. In gray matter, it is estimated that housekeeping like synthesis of proteins and other molecules use just a quarter of the energy, while the rest is consumed by Na,K-ATPases (Attwell and Laughlin, 2001).

## Cardiotonic steroids

The vital importance of the Na,K-ATPase for animals makes it a natural target for toxins produced by plants or animals that want to avoid being eaten. At least 12 different plant families and several species of the *Bufo* toads produce Na,K-ATPase inhibitors, the so-called cardiotonic steroids (Gao et al., 2011). Plant cells have no endogenous Na,K-ATPase, since they use a proton gradient to energize their membranes, so Na,K-ATPase inhibitors are not toxic to them.

Several insect species feed on plants with cardiotonic steroids and store the toxins to make themselves poisonous (Zhen et al., 2012). The poisonous insects and toads can, nonetheless, become prey to other animals, including reptiles, hedgehogs, and rodents. All animals that produce or ingest cardiotonic steroids tolerate the toxin because of specific mutations in their Na,K-ATPase encoding genes that make the pumps insensitive to the inhibitor (Ujvari et al., 2015), a classical example of an evolutionary arms race, where one species develops a weapon, and then its predator develops a defense against it.

Cardiotonic steroids like digitalis from the foxglove plant have been used to treat heart conditions for centuries. They are recommended for atrial fibrillation and reduce hospital admission for heart failure patients, but have not been shown to affect mortality rates (Ziff and Kotecha, 2016). The mechanism-of-action of digitalis is still a matter of debate. It has been believed that inhibition of the Na,K-ATPase in cardiomyocytes raises cytoplasmic sodium levels, thereby inhibiting the sodium-calcium exchanger and raising cytoplasmic calcium levels, which is imagined to have a direct effect on the heart's contractility, but there is also evidence to suggest that digitalis strengthens the parasympathetic nervous system by vagal activation and slows heart rate (Ziff and Kotecha, 2016).

At non-saturating levels (5–10 nM), cardiotonic steroids have been suggested to act as ligands and the Na,K-ATPase as a receptor that initiates intracellular signaling via e.g., the inositol 1,4,5-triphosphate receptor and the Src kinase to promote cellular proliferation. In the body, cardiotonic steroids may arise from medication, but low levels of endogenous cardiotonic steroids have also been measured (Aperia et al., 2016). It remains controversial what the physiological significance of Na,K-ATPase signaling may be, and transcriptome differences in response to cardiotonic steroids were only seen if the relative intracellular concentrations of sodium and potassium changed, i.e., if there was a direct effect on the ion pumping mechanism (Klimanova et al., 2017).

## The basic mechanism of ion transport

Cardiotonic steroids bind the Na,K-ATPase from the extracellular side in the suggested ion exchange pathway as revealed by the crystal structure of a high-affinity binding complex between the cardiotonic steroid digoxin and Na,K-ATPase purified from pig kidney (Laursen et al., 2015, Figure 1). The complex contains three protein subunits, namely the ten transmembrane (TM) helix alpha subunit and the single TM beta and FXYD subunits. The beta subunit has a large, glycosylated extracellular part, and for the smaller FXYD subunit, only the TM part is resolved in the structure. The alpha subunit has three cytoplasmic domains, the nucleotide binding (N), the phosphorylation (P) and the actuator (A) domains, which function as the kinase, the substrate and the phosphatase, respectively, in the catalytic cycle, when ATP is hydrolyzed (Figure 2). A conserved aspartate in the P-domain is phosphorylated and dephosphorylated during the cycle, and the movements of the cytoplasmic domains cause the connected TM helices to bind and release the pump's substrates in response. The Na,K-ATPase was the founding member of the P-type ATPase family (Skou, 1957) whose members share this basic mechanism to transport numerous different cations and even lipids. In addition to the ouabain-bound Na,K-ATPase, crystal structures of two major steps in the catalytic cycle have been solved, namely of the potassium (Morth et al., 2007; Shinoda et al., 2009) and sodium (Kanai et al., 2013; Nyblom et al., 2013) occluded forms (Figure 2).

**Figure 1:**
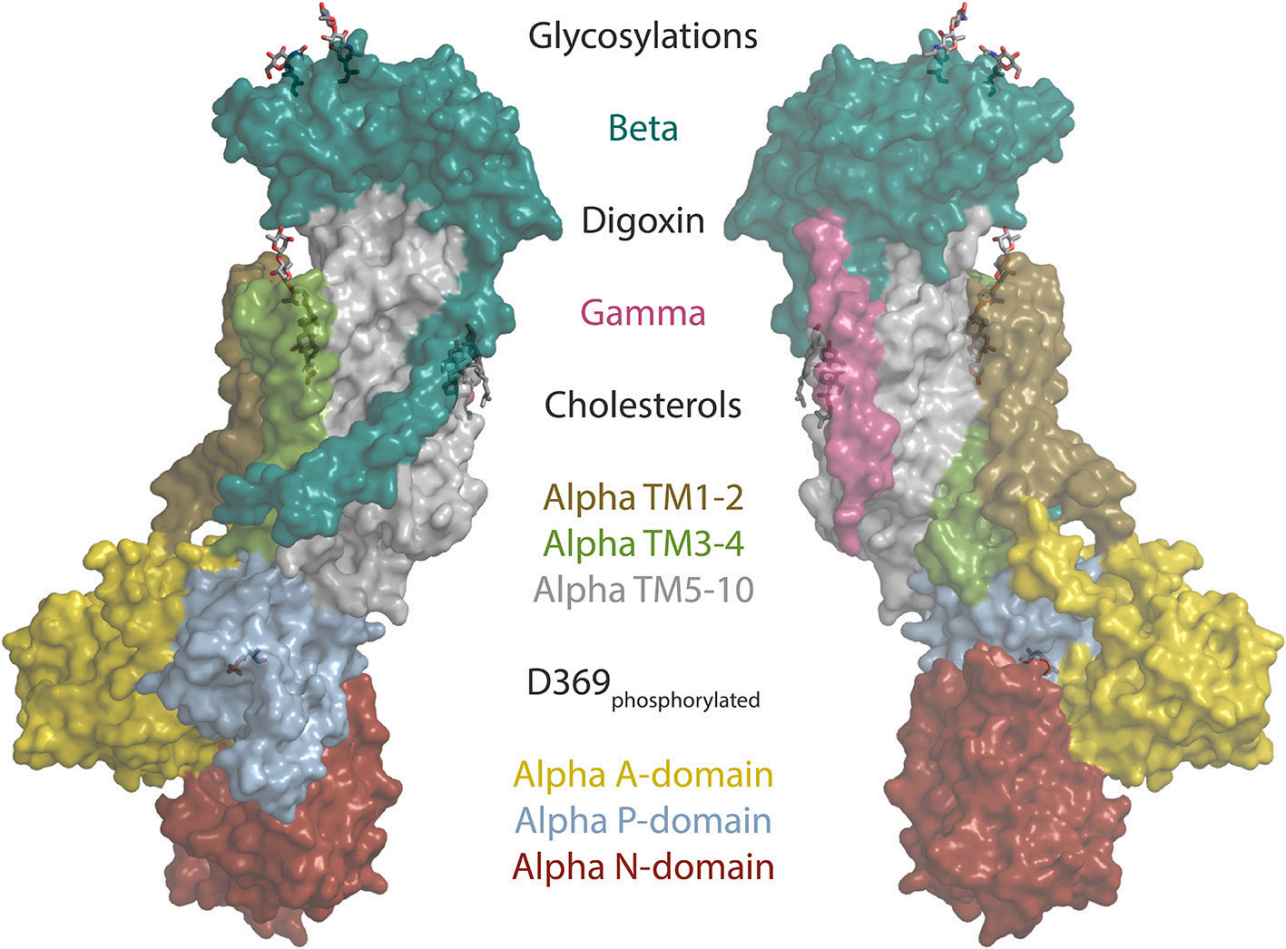
The structure of the sodium pump. Surface representation of the digoxin bound alpha1 isoform structure from pig (Laursen et al., 2015) (PDB ID: 4RET). Sodium pump subunits and domains are shown in colors as indicated. The two beta glycosylations, digoxin, two cholesterols and the phosphorylated aspartate (D369) are shown as sticks.

**Figure 2:**
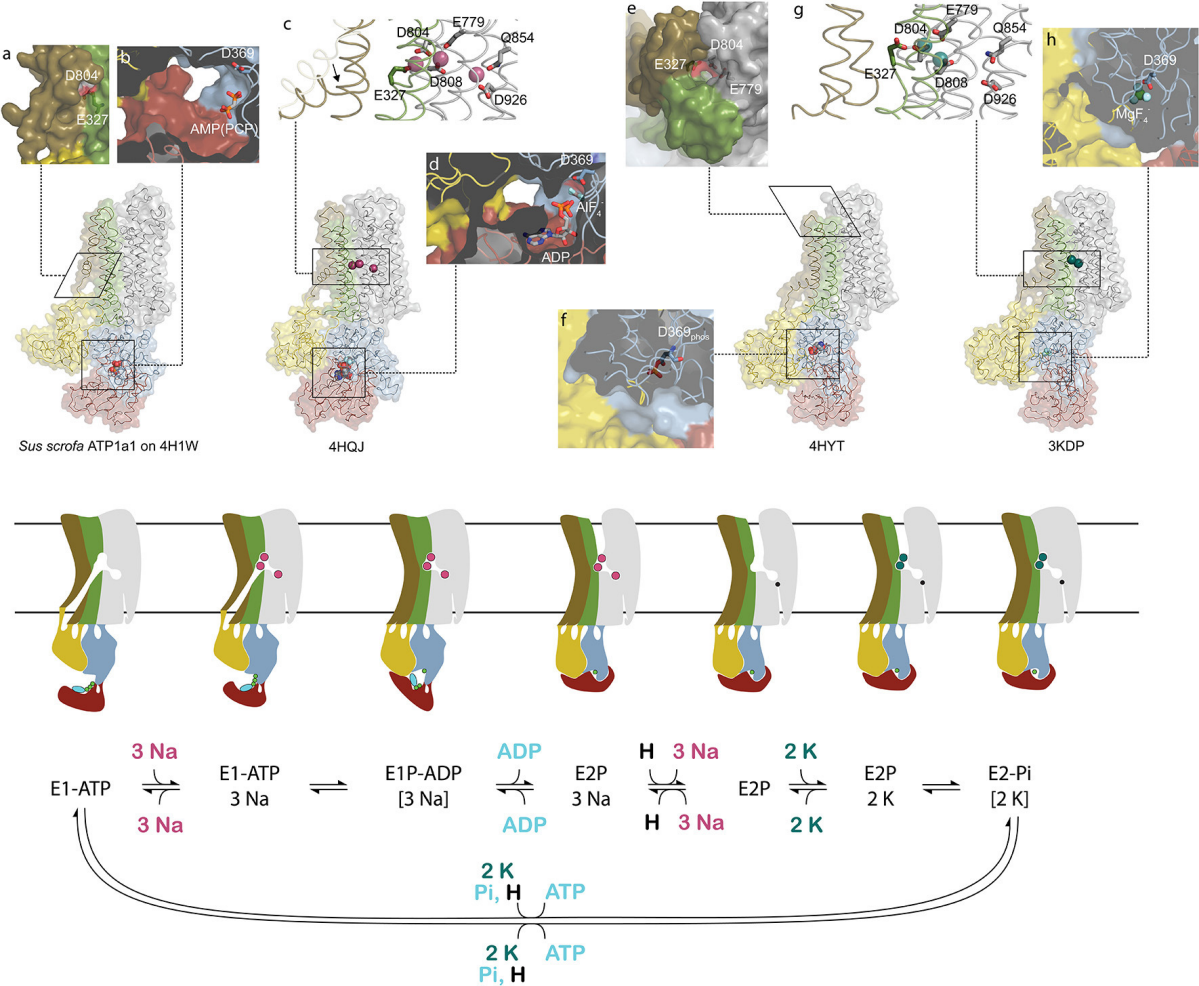
Conformational changes during the sodium pump catalytic cycle. Three sodium pump structures and a homology model are positioned in accordance with the catalytic cycle shown below as both cartoon and reaction scheme. The eight inserts labeled with small letters highlight important structural details. The homology model of pig alpha1 on the SERCA E1-ATP state (Winther et al., 2013) (PDB ID: 4H1W) shows an inwardly opened conformation with access to the ion-binding sites, here visualized by D804 and E327 **(a)**. In the structure, only the beta and gamma phosphates of the non-hydrolysable ATP analog AMPPCP are resolved and demonstrate a non-primed positioning for reaction with D369 **(b)**. After binding of three sodium ions, TM1 rearranges to a position that blocks the cytoplasmic entrance pathway (arrow in **c**), and the cytoplasmic domains tighten around the nucleotide that reacts with D369 **(d)**. Following sodium occlusion ADP is released and an extracellular pathway allows the exit of the three sodium ions. In the externally opened conformation, here imitated by the ouabain bound structure 4HYT shown without the inhibitor, three ion-binding residues are directly visible from the outside **(e)**, and the intracellular domains are completely wrapped around the phosphorylated D369 **(f)**. Binding of two extracellular potassium ions **(g)** initiates closure of the extracellular gate and dephosphorylation of D369 **(h)**. The narrow pathway from the cytoplasm to the sodium specific binding site in the cartoon representation shows the proposed C-terminal proton path utilized for charge conservation. Color coding as in Figure 1.

The transport of ions against their concentration gradients requires that the transmembrane ion binding site acts like a space shuttle airlock with gates on either side of which at least one is always locked to avoid the energetically favored flow of ions in the opposite direction. When the Na,K-ATPase opens toward the cytoplasm, TM1 is believed to slide up (as in the related calcium pump SERCA, Winther et al., 2013) and allow three sodium ions to access the high-affinity binding sites in the middle of the membrane, where negative charges on aspartate and glutamate residues compensate for the charges of the positively charged ions (Figure 2). When bound, the inner gate closes to form an occluded pump, and the P-domain is phosphorylated by ATP. The pump is said to be in the E1 form when it has high affinity for sodium, and in the E2 form when it has high affinity for potassium. The transition from phosphorylated E1 (called E1P) to E2P is coupled to release of ADP, opening of the outer gate and release of the three sodium ions to the extracellular side. It is proposed that a proton from the cytoplasm promotes the sodium release and compensates for negative charge at the ion binding site unique for sodium, the so-called site III, when sodium is released (Poulsen et al., 2010). Potassium can then bind from the extracellular side, the outer gate closes to occlude the two potassium ions, and the aspartate in the P-domain is dephosphorylated. Binding of ATP, a transition back to the E1 form, and opening of the inner gate lead to cytoplasmic release of the proton in site III and of the potassium ions in sites I and II, and the pump is ready for another cycle (Figure 2).

## Isoform expression, function and physiology

**Figure 3:**
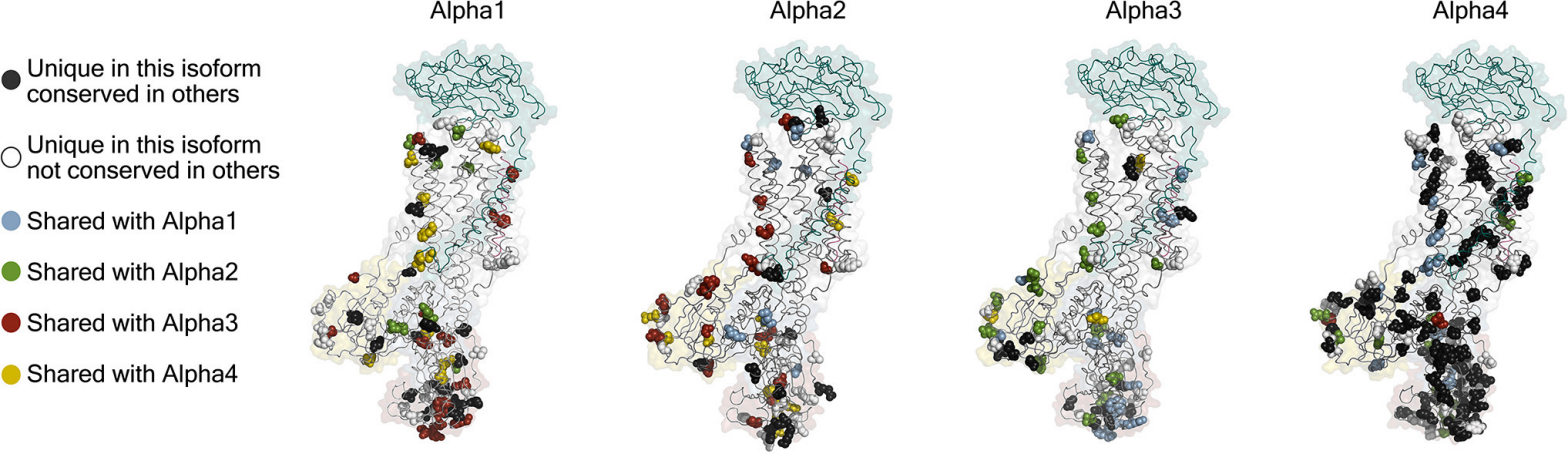
Homology models of the four human alpha isoforms. Models were built from the potassium occluded 3KDP structure (Morth et al., 2007), and the isoform differences are highlighted as spheres. Conservative differences are not included which means that the following groups of amino acids were treated as identical (L, I, V), (E, D), (K, R), (Q, N), (S, T), (Y, F), and (M, C) while H, G, P, A, and W were ungrouped.

The first indication that there is more than one isoform of the Na,K-ATPase subunits came from titration with ouabain on mouse brain preparations, which showed a biphasic curve of ATPase activity (Marks and Seeds, 1978). It was later found that the biphasic curve, which is also found in rat, is due to a specific reduction in ouabain affinity (low mM) of alpha1 in rodents that makes it easily distinguished from the two high affinity isoforms found in brain, alpha2, and alpha3 (low μM affinities). The primary sequences of the alpha isoforms are well conserved: alpha1, 2, and 3 are about 87% identical to each other and about 78% identical to the sperm-specific alpha4 (Shamraj and Lingrel, 1994). Between species, the identity percentages of alpha1, 2, and 3 are in the high nineties and for alpha4 in the low eighties (Clausen et al., 2011). Mapping the isoform differences on homology models of each alpha (Figure 3) shows that the variation is generally found at the surface of the protein, while the ion binding interior of the membrane domain and the linkers to the cytoplasmic domains are highly conserved. The most diverse part of the protein is the surface of the N-domain, especially in alpha4. The basic function of the pump will depend most strongly on the interior parts where ions are transported in response to the ATP hydrolysis, while differences on the surface allow each isoform to have its own protein-protein interaction networks.

In addition to the four alpha isoforms, mammals express three beta and seven FXYD subunit isoforms. The combination of alpha1 and beta1 is expressed most widely (table), and any alpha can be expressed with any beta to yield a functional pump in *Xenopus* oocytes (Crambert et al., 2000; Hilbers et al., 2016), though *in vivo* the associations may be more selective (Tokhtaeva et al., 2012; Habeck et al., 2016).

The different isoforms have different kinetic properties and affinities. Depending on the assay used, the details can vary, but for example for the alphas, there is general consensus that alpha1 has relatively high apparent potassium affinity, and alpha3 relatively low sodium affinity (Blanco, 2005). The beta and FXYD subunits further affect the functional properties (Arystarkhova and Sweadner, 2016; Hilbers et al., 2016), so the different subunits allow cells to have Na,K-ATPase activity with optimized functional characteristics. In addition, the subunits differ in how they are trafficked to and localized in the membrane, which posttranslational modifications they are subject to, and importantly what cellular partners they interact with. Therefore, the distinct expression profiles of the Na,K-ATPase subunits enable fine-tuning in time and tissues of the pumping activity (Table).

Since the ionic gradients are of vital importance for any organ, disturbance of the Na,K-ATPase activity has been implicated in many pathophysiological conditions, including cancer (Durlacher et al., 2015), diabetes (Vague et al., 2004), and heart failure (Schwinger et al., 2003), and the pump has been suggested as a potential chemotherapy target (Alevizopoulos et al., 2014), though evidence is still lacking for its efficacy (Durlacher et al., 2015).

## Alpha1

The alpha1 subunit is essentially omnipresent at the tissue and cellular levels. One organ that relies heavily on sodium and potassium gradients is the heart, where the rhythmic action potentials and accompanying calcium fluxes determine the muscle contractions. All animal hearts examined express alpha1, while it varies between species if it is the only isoform or if alpha2 and/or alpha3 can be detected (Sweadner et al., 1994; McDonough et al., 2002; Dostanic et al., 2004; Henriksen et al., 2013, Table). In human hearts, all three isoforms are expressed, and in failing human hearts the protein levels of alpha1 and alpha3 are reduced 30–40% in the left ventricle compared to non-failing hearts (Schwinger et al., 1999), and at the RNA level, the left and right ventricles of failing hearts show around 50% reduction in transcripts encoding alpha1 and alpha2 (Borlak and Thum, 2003). An increase in the intracellular sodium concentration due to lower levels of Na,K-ATPase will likely decrease calcium export by the Na^+^/Ca^2+^-exchanger, which may be the main extrusion pathway in cardiomyocytes. The downregulation of Na,K-ATPases is also a probable reason for higher sensitivity to cardiotonic steroids in failing hearts (Shamraj et al., 1993).

Several physiological processes depend on a very strict regulation of the subcellular localization of alpha1. Cavities and surfaces in the body are lined by sheets of polarized epithelial cells that have distinct membrane domains; an apical membrane that faces the lumen and a basolateral membrane that is oriented away from the lumen. Depending on the physiological role of an epithelial sheet, the cellular sorting machinery makes sure that alpha1 ends up in either the apical or the basolateral membrane.

When the intention is to minimize bodily loss of sodium through e.g., urine and sweat, alpha1 is sent to the basolateral membrane of the epithelial cells as seen in the renal tubular system (Caplan et al., 1986) and in the secretory coils of sweat glands (Zhang et al., 2014), where sodium reuptake is driven by channels and secondary transporters.

To maintain a low level of potassium while indirectly supplying water to the cerebrospinal fluid of the central nervous system, the epithelial cells that line the choroid plexus sort alpha1 to the apical membrane (Gundersen et al., 1991; Brown et al., 2004). The same strategy of apical alpha1 sorting is utilized in the eye where retinal pigment epithelium supplies the fluid of the subretinal space with the high sodium concentration needed to maintain the dark current that keeps photoreceptor cells depolarized in the absence of light (Miller et al., 1978; Sparrow et al., 2010).

## Alpha2

The alpha2 isoform is predominantly expressed in muscle (heart and skeletal) and brain (in astrocytes and glia cells). It is noteworthy that while astrocytes co-cultured with neurons readily express alpha2, purified astrocytes grown without neurons only rarely express it (Peng et al., 1998). With a relatively high sensitivity to voltage (Crambert et al., 2000; Horisberger and Kharoubi-Hess, 2002; Larsen et al., 2014; Clausen et al., 2016) and in combination with beta2, a remarkably reduced affinity for potassium (K^+^ K_0.5_ 3 mM, other isoforms have K_0.5_ ~ 1 mM, Crambert et al., 2000; Larsen et al., 2014), the alpha2beta2 complex in glia and astrocytes seems perfectly geared for clearance of potassium after intense neuronal activity, being maximally active when the potassium concentration is high, and the membrane potential depolarized.

In the heart, alpha2 preferentially assembles with beta2 and specifically localizes to the T-tubular membranes, while alpha1beta1 is more uniformly distributed in both T-tubular membranes and external sarcolemma membranes (Habeck et al., 2016). The alpha2 subunit localizes close to the Na^+^/Ca^2+^-exchanger in contractile tissue and may thereby indirectly serve to assist the regulation of calcium levels (Juhaszova and Blaustein, 1997). Similar to the suggested role in glia cells, the high voltage sensitivity and low potassium affinity of alpha2beta2 are proposed to ensure that additional Na,K-ATPase activity is available during the long-lasting cardiac action potential (Stanley et al., 2015; Habeck et al., 2016). In skeletal muscle, the alpha2 activity is rapidly controlled in response to changes in muscle use, suggesting that it may be adapted to reacting on dynamic changes in muscle activity (Kravtsova et al., 2016).

## Alpha3

Alpha3 is highly expressed in the brain with a main localization in neuronal projections (Bottger et al., 2011) and to some extend in dendritic spines (Kim et al., 2007; Blom et al., 2016). During intense neuronal activity, the concentration of sodium in dendrites and spines can increase dramatically, estimates as high as 100 mM have been proposed (Rose and Konnerth, 2001), and the required clearance of intracellular sodium is mainly attributed to alpha3 (Azarias et al., 2013), which has relatively low sodium affinity, 25–50 mM, compared to Na^+^ K_0.5_ of approximately 10 mM for other isoforms (Zahler et al., 1997; Blanco and Mercer, 1998; Crambert et al., 2000). The alpha3-containing pumps in neurons thus appear to be optimized for high intensity neuronal firing.

Interestingly, several studies of disease mechanisms have found that neurodegenerative effects may be caused by direct interactions with alpha3. In Alzheimer's disease, amyloid-beta can cause neurodegeneration, and the same is true for alpha-synuclein in Parkinson's disease. Amyloid-beta as well as alpha-synuclein assemblies were found to interact with alpha3, and mapping of the interaction domain located a specific extracellular loop in the pump as the target in both cases (Ohnishi et al., 2015; Shrivastava et al., 2015). Furthermore, misfolded SOD1 protein, which is associated with amyotrophic lateral sclerosis, can bind an intracellular domain of alpha3 and inhibit the pump function (Ruegsegger et al., 2016).

Alpha3 is also highly expressed in the human heart; interestingly with gender-specific differences: the relative expression of alpha3 to alpha1 is several-fold higher in men than in women as judged from RNA levels (Gaborit et al., 2010).

## Alpha4

Spermatozoa are exceedingly specialized cells unique in their dependence on being able to thrive and carry out complex tasks inside other organisms. Furthermore, they are subject to a more profound evolutionary pressure than other cells, because only a single spermatozoon from a batch of hundreds of millions has a chance of fertilizing an egg, and only mutations that make better spermatozoa count in this race. The competitive spermatozoa evolution is evident from the high number of unique sperm cell proteins, particularly membrane proteins (Dorus et al., 2010). One of the sperm specific proteins is the alpha4 isoform of the sodium pump (Hlivko et al., 2006; McDermott et al., 2012), and the strong evolutionary pressure on sperm proteins is very apparent for alpha4, which is the most divergent of the Na,K-ATPase alpha subunits, both when compared with the other three alpha isoforms (Figure 3), and when compared between different species (Clausen et al., 2011). While alpha4 is considered a sperm specific Na,K-ATPase, a smaller protein from human skeletal muscle cross react with an alpha4 antisera (Sugiura et al., 2005), and mRNA that hybridize with alpha4 probes are present in human and mouse skeletal muscle (Keryanov and Gardner, 2002).

Although sperm cells express alpha1 in addition to alpha4, male mice are completely sterile if they lack alpha4, and their spermatozoa are unable to fertilize eggs *in vitro* although their viability is unaffected (Jimenez et al., 2011a). Lack of alpha4 reduces sperm motility, depolarizes the membrane potential and increases intracellular sodium (Jimenez et al., 2011a), whereas overexpression of alpha4 increases their motility (Jimenez et al., 2011b; McDermott et al., 2015).

At the biophysical level, when compared with the other isoforms in a cell free system, alpha4 has low K_0.5_ for ouabain and sodium, and regular for potassium (Blanco et al., 1999). When studied in a cellular system, alpha4 is less affected by changes in voltage, extracellular sodium and temperature than alpha1 (Clausen et al., 2016).

If greater evolutionary pressure has optimized alpha4 to increase the probability that spermatozoa reach and fertilize an egg in the female oviduct, there could be specific parameters that make this pump more suitable for the task than the other isoforms. Interestingly it has been demonstrated that as spermatozoa capacitate in the female genital tract, the catalytic activity of alpha4 is up-regulated due to an increased availability of pumps in the membrane (Jimenez et al., 2012), suggesting that alpha4 supports the hyperpolarization and decreased intracellular sodium concentration characteristic of capacitated spermatozoa. An alternative, and not necessarily conflicting, hypothesis is that since pumps in the membrane of spermatozoa experience dramatic changes in e.g., extracellular sodium levels and membrane potential as the cell passes from testis to oviduct, it could be advantageous with a pump that, like alpha4, responds less to environmental alterations (Clausen et al., 2016).

## Betas

The Na,K-ATPase beta subunit is part of the functional core of the pump and is required for its trafficking to the plasma membrane. The sequence identity of the three human isoforms are 39% (beta1 and 2), 36% (beta1 and 3), and 47% (beta2 and 3). It has a small (30 amino acid) N-terminal, intracellular domain, a TM helix, and a larger (~240 amino acids) C-terminal, extracellular domain. Like the alpha isoforms, the different beta isoforms have distinct tissue and cell-type specific expression profiles (Table 1). The beta2 isoform was originally found in glia where it is involved in cell-cell contacts and hence it was initially called Adhesion Molecule On Glia (AMOG, Antonicek et al., 1987; Martin-Vasallo et al., 1989; Gloor et al., 1990).

**Table 1:** Protein expression in tissues from the indicated mammals detected by western blots (WB) or immunostaining (IS) of Na,K-ATPase alpha1 (a1), alpha2 (a2), alpha3 (a3), alpha4(a4), beta1(b1), beta2(b2), beta3(b3), and FXYD(g). Cultured cells not included.

**Organ/part**	**Tissue/structure**	**Cell/tubule type**	**Protein found**	**Protein probed for**	**Organism**	**Method**	**References**
Brain	Microvessels		a1, a2, a3, b1, b2	a1, a2, a3, b1, b2	rat	WB	Zlokovic et al., 1993
Brain	Choroid plexus		a1, b1, b2	a1, a2, a3, b1, b2	rat	WB	Zlokovic et al., 1993
Brain	Several areas are extensively studied	Only neurons	a3	a3	mouse	IS	Bottger et al., 2011
Brain				a4	human	WB	Hlivko et al., 2006
Brain	Several areas are studied	Neurons and Astrocytes	b1, b2	b1, b2	rat	WB, IS	Lecuona et al., 1996
Brain	Cerebral cortex	Astroglia	a2	a2	rat	IS	Cholet et al., 2002
Brain	Axolemma, cerebrum, cerebellum, corpus callosum, optic nerve		a1, a2, a3	a1, a2, a3	rat	WB	Urayama et al., 1989
Brain	Microsomes		b3	b3	rat	WB	Arystarkhova and Sweadner, 1997
Colon	Mucosae, submucosae	Epithelial cells, mesenchymal cells	a1, a3, b1, b2	a1, a3, b1, b2	human	IS	Baker Bechmann et al., 2016
Colon	Myenteric plexus	Neurons	(a1), a3, b1, (b2)	a1, a3, b1, b2	human	IS	Baker Bechmann et al., 2016
Colon	Myenteric plexus	Glia cells	(a1), (a3), b1, b2	a1, a3, b1, b2	human	IS	Baker Bechmann et al., 2016
Colon	Muscularis propia	Smooth muscle cells	(a1), a3, b2	a1, a3, b1, b2	human	IS	Baker Bechmann et al., 2016
Erythrocytes			a1, a3, b1, b2, b3	a1, a2, a3, b1, b2, b3	human	WB	Hoffman et al., 2002
Eye	Pars plicata	Nonpigmented epithelium	a1, a2, a3	a1, a2, a3	bovine	IS	Ghosh et al., 1990
Eye	Pars plicata	Pigmented epithelium	a1	a1, a2, a3	bovine	IS	Ghosh et al., 1990
Eye	Pars plana	Nonpigmented epithelium	a1, a2	a1, a2, a3	bovine	IS	Ghosh et al., 1990
Eye	Pars plana	Pigmented epithelium	a1	a1, a2, a3	bovine	IS	Ghosh et al., 1990
Eye	Retina	Photoreceptors	a3, b2, b3	a1, a2, a3, b1, b2, b3	mouse	IS	Wetzel et al., 1999
Eye	Retina	Horizontal cells	a1, a3, b1	a1, a2, a3, b1, b2, b3	mouse	IS	Wetzel et al., 1999
Eye	Retina	Bipolar cells	a3, b2	a1, a2, a3, b1, b2, b3	mouse	IS	Wetzel et al., 1999
Eye	Retina	Ganglion cells	a1, a3, b1, b2	a1, a2, a3, b1, b2, b3	mouse	IS	Wetzel et al., 1999
Eye	Retina	Amacrine cells	a3, b1	a1, a2, a3, b1, b2, b3	mouse	IS	Wetzel et al., 1999
Eye	Retina	Müller cells	a1, a2, b2	a1, a2, a3, b1, b2, b3	mouse	IS	Wetzel et al., 1999
Eye	Retina	Pigmented epithelium	a1, b1	a1, a2, a3, b1, b2, b3	mouse	IS	Wetzel et al., 1999
Heart	Ventricular myocardium		a1, a2	a1, a2, a3	rat	WB	Sweadner et al., 1994
Heart	Ventricular myocardium		a1, a2, a3	a1, a2, a3	human	WB	Sweadner et al., 1994
Heart	Left ventricle, right ventricle, atrium, ventricular septum, papillary muscle, aorta		a1, a3	a1, a2, a3	macaque	WB	Sweadner et al., 1994
Heart		Cardiomyocytes	a1, a2, b1, b2, b3	a1, a2, b1, b2, b3	mouse/rat	WB, IS	Habeck et al., 2016
Heart	Microsomes		b3	b3	rat	WB	Arystarkhova and Sweadner, 1997
Inner ear	Cochlea	External sulcus cells, GER cells, Root cells, Interdental cells, Claudius cells, Reissner's membrane cells, Spiral ligament fibrocytes type IV	a1, b1	a1, a2, a3, b1, b2	rat	IS	Peters et al., 2001
Inner ear	Cochlea	Marginal cells	a1, b1, b2	a1, a2, a3, b1, b2	rat	IS	Peters et al., 2001
Inner ear	Cochlea	Suprastrial fibrocyes, Spiral ligament fibrocytes type II	a1, a2, b1	a1, a2, a3, b1, b2	rat	IS	Peters et al., 2001
Inner ear	Cochlea	Coclear neurons	a1, a3, b1	a1, a2, a3, b1, b2	rat	IS	Peters et al., 2001
Inner ear	Vestibulum	Supporting cells, transitional cells	a1, b1	a1, a2, a3, b1, b2	rat	IS	Peters et al., 2001
Inner ear	Vestibulum	Nonsensory cells, Dark cells	a1, b1, b2	a1, a2, a3, b1, b2	rat	IS	Peters et al., 2001
Inner ear	Vestibulum	Vestibular neurons	a1, a3, b1	a1, a2, a3, b1, b2	rat	IS	Peters et al., 2001
Inner ear	Endolymphatic sac/duct	Endolymphatic sac cells, endolymphatic duct cells	a1, b1	a1, a2, a3, b1, b2	rat	IS	Peters et al., 2001
Inner ear	Spiral ganglion, Organ of Corti	Type I spiral ganglion neurons	(a1), a3	a1, a2, a3	rat	IS	McLean et al., 2009
Inner ear	Spiral ganglion, Organ of Corti	Type II spiral ganglion neurons	(a1)	a1, a2, a3	rat	IS	McLean et al., 2009
Inner ear	Spiral ganglion, Organ of Corti	Phalangeal cells	a1	a1, a2, a3	rat	IS	McLean et al., 2009
Joints	Cartilage	Chondrocytes	a1, a3, b1, b2	a1, a2, a3, b1, b2	bovine	WB, IS	Mobasheri et al., 1997
Kidney	Renal medulla		a1, a3	a1, a2, a3	rat	WB	Urayama et al., 1989
Kidney	Nephron	Proximal convoluted tubule, proximal straight tubule, medullary thick ascending limb, distal convoluted tubule, connecting tubule	a1, b1, g	a1, b1, g	rat	IS	Wetzel and Sweadner, 2001
Kidney	Nephron	Cortical thick ascending limb, cortical collecting duct	a1, b1	a1, b1, g	rat	IS	Wetzel and Sweadner, 2001
Kidney	Nephron	Glomeruli, thin limbs of henle, medullary collecting duct		a1, b1, g	rat	IS	Wetzel and Sweadner, 2001
Kidney	Microsomes		b3	b3	rat	WB	Shyjan and Levenson, 1989
Kidney				a4	human	WB	Hlivko et al., 2006
Liver			a1	a1, a2, a3, b1	rat	WB	Shyjan and Levenson, 1989
Liver			b3	b3	rat	WB	Arystarkhova and Sweadner, 1997
Liver		Hepatocytes, ephitelial cells	a1, b1, b2	a1, a3, b1, b2	human	IS	Baker Bechmann et al., 2016
Liver		Endothelial cells		a1, a3, b1, b2	human	IS	Baker Bechmann et al., 2016
Lung			b3	b3	rat	WB	Arystarkhova and Sweadner, 1997
Lung			a1, a2	a1, a2, a3, b1	rat	WB	Shyjan and Levenson, 1989
Lung			b1	b1	rat	WB	Zhang et al., 1997
Placenta			a1, a2, a3	a1, a2, a3	human	WB	Esplin et al., 2003
Prostate		Epithelial cells	a1, b1, b2, b3	a1, a2, a3, b1, b2, b3, g	rat	IS	Mobasheri et al., 2003
Prostate		Smooth muscle, stroma	a1, a2, b1, b2	a1, a2, a3, b1, b2, b3, g	rat	IS	Mobasheri et al., 2003
Skeletal muscle	Extensor digitorum longus		a1, a2, b1, b2, b3	a1, a2, a3, b1, b2, b3	mouse	WB	He et al., 2001
Spleen	Microsomes		a1	a1, a2, a3, b	rat	WB	Shyjan and Levenson, 1989
Testis		Sperm	a1, a4	a1, a4	rat	IS	Blanco et al., 2000
Testis		Sperm	a4	a4	human	WB, IS	Hlivko et al., 2006
Testis		Sperm	a4	a4	bovine	WB, IS	Newton et al., 2009
Testis		Sperm	a1, a4	a1, a4	mouse	WB	Jimenez et al., 2011a
Testis	Microsomes		b3	b3	rat	WB	Arystarkhova and Sweadner, 1997
Uterus		Epithelial cells, smooth muscle cells	a1, a2, (a3), b1, b2, (b3), g	a1, a2, a3, b1, b2, b3, g	human	IS	Floyd et al., 2010

*Shyjan and Levenson, 1989; Urayama et al., 1989; Ghosh et al., 1990; Zlokovic et al., 1993; Sweadner et al., 1994; Lecuona et al., 1996; Arystarkhova and Sweadner, 1997; Mobasheri et al., 1997, 2003; Zhang et al., 1997; Wetzel et al., 1999; Blanco et al., 2000; He et al., 2001; Peters et al., 2001; Wetzel and Sweadner, 2001; Cholet et al., 2002; Hoffman et al., 2002; Esplin et al., 2003; Hlivko et al., 2006; McLean et al., 2009; Newton et al., 2009; Floyd et al., 2010; Bottger et al., 2011; Jimenez et al., 2011a; Baker Bechmann et al., 2016; Habeck et al., 2016*.

There are three conserved disulfide bonds in the extracellular domain, which are important for forming a stable pump (Noguchi et al., 1994), and the domain has three, eight, and two glycosylation sites in beta1, 2, and 3, respectively. Removal of the glycosylations causes retention in the endoplasmatic reticulum of beta2, but not of beta1 or 3, suggesting that the glycosylations play individual roles in the different isoforms (Tokhtaeva et al., 2010).

Beta1 may further respond to oxidative stress by glutathionylation of a cysteine in the middle of its transmembrane helix, a cysteine not found in the other betas (Rasmussen et al., 2010). *In vitro* studies have shown that the E1 state of the enzyme favors the cysteine to be glutathionylated more than the E2 state (Garcia et al., 2015).

Functionally, beta2 has the strongest effects on the kinetic properties of the pump, reducing the apparent potassium affinity and raising the extracellular sodium affinity compared to beta1 and 3 (Larsen et al., 2014). This effect of beta on the pump was found to be due to the TM helix rather than the N- or C-terminal domains, and specifically to a difference between the three isoforms in the tilt angles of their TM helices (Hilbers et al., 2016).

The different beta isoforms and the variation in their post-translational modifications facilitate regulated Na,K-ATPase activity, adapted to different tissues and to environmental changes.

In mouse heart, the major beta isoform is beta1. Nonetheless, specific inactivation of its gene in cardiomyocytes results in a phenotype that appears healthy until approximately 10 months of age, and reduced contractility and enlarged hearts are only found after an additional 3–4 months (Barwe et al., 2009). Interestingly, heterozygous knock-out of alpha1 also results in reduced contractility, while heterozygous knock-out of alpha2 results in hyper contractility (James et al., 1999); the alpha2 phenotype may, however, be a secondary effect of alpha2 deficiency in the brain (Rindler et al., 2013). Beta2 knock-out mice succumb at day 17–18 after birth, possibly due to dysfunction of vitally important brain structures (Magyar et al., 1994). There are currently no confirmed human genetic diseases linked with mutations in any of the beta subunits.

## FXYDs

The minimal functional unit of the Na,K-ATPase has an alpha and a beta subunit, but it can be further modified by a third subunit, the FXYD, named after a shared PFxYD motif in the N-terminal, extracellular part of the single TM protein. Mammals express seven FXYD proteins, most of which appear to lower the substrate affinities or Vmax of the pump, though they may also serve functions in addition to modulating Na,K-ATPase function (Geering, 2005).

FXYD1 is highly expressed in the heart, skeletal muscle and brain. Two serines in the intracellular part can be phosporylated by protein kinases A and C, and its alternative name, phospholemman, reflects that it was originally described as a highly phosphorylated (~15–45%) heart protein (Palmer et al., 1991; Walaas et al., 1994; Cheung et al., 2010). Phosphorylation decreases the inhibitory effect of FXYD1 (Cheung et al., 2010; Mishra et al., 2015), and mouse knock-out of the subunit increases Na,K-ATPase activity in the heart (Jia et al., 2005; Bell et al., 2008).

FXYD2, or gamma, was the first FXYD found to be associated with the Na,K-ATPase (Forbush et al., 1978). It is highly expressed in the kidney, and like FXYD1, lowers pump activity, an inhibition that is also relieved if the protein is knocked out in mice (Jones et al., 2005; Arystarkhova, 2016). Mice lacking FXYD2 are viable, but have impaired reproduction, possibly because of a metabolic phenotype where glucose is highly tolerated (Arystarkhova et al., 2013). In the collecting duct of the kidney, FXYD4 is expressed, which, unlike most FXYDs, increases the pump's sodium affinity and thus enhances activity (Geering, 2005).

The roles of FXYD3 (Mat-8) and FXYD5 (dysadherin) are unclear, but they appear to be overexpressed in some cancer cells (Arimochi et al., 2007; Nam et al., 2007), and FXYD6 and 7 are expressed in the brain (Geering, 2005). There are currently no confirmed human genetic diseases linked with mutations in FXYD subunits.

## Disease-causing mutations in Na,K-ATPase alpha isoforms

### Alpha1 in disease

Deleterious mutations in *ATP1A1* are unlikely to be compatible with life, but in a subset of aldosterone producing adenomas (APAs) in the adrenal gland, somatic mutations in *ATP1A1* can contribute to the altered hormone balance (Azizan et al., 2013; Beuschlein et al., 2013). Adrenal overproduction of aldosterone is the cause of hypertension in up to 10% of hypertensive patients, and if adrenal adenomas are identified and removed, the patients will typically be cured.

The normal signal pathway is that adrenal cells respond to the peptide hormone angiotensin II and to extracellular potassium by depolarization and opening of voltage-gated calcium channels, and the rise in cytoplasmic calcium levels stimulates expression of the aldosterone synthase. The dependence on extracellular stimuli can, however, be circumvented if the downstream signals are directly induced by mutations in the systems that normally control membrane potential and calcium levels, including a potassium channel (*KCNJ5*, Choi et al., 2011), a calcium channel (*CACNA1D*, Azizan et al., 2013), and a calcium pump (*ATP2B3*, Beuschlein et al., 2013). Most recently, a link was found between APAs and mutations causing beta-catenin (*CTNNB1*) to excessively activate the Wnt-signaling pathway that normally controls adrenocortical development (Teo et al., 2015). Even in adrenal glands with hyperplasia but no adenoma, somatic mutations in *CACNA1D* (Scholl et al., 2015) and *ATP1A1* (Nishimoto et al., 2015) have been found.

The coupling of *ATP1A1* mutations to hypertension does not immediately make as much sense as for the potassium channel and calcium regulators. Why would a single non-functional copy of the gene have such marked effect on aldosterone production? One clue was that there is a prominent hotspot for mutation in TM1 next to the ion coordinating Glu327 in TM4, especially Leu104Arg is commonly found in APAs, and another reoccurring spot for alterations is in TM4 right next to Glu327 (Figure 1, Kopec et al., 2014). A third hotspot is in TM9, where deletions close to site III, the sodium-specific site, are found (Figure 2). Expression of the mutant forms of the pump cause depolarization (Beuschlein et al., 2013; Stindl et al., 2015), and expression studies in *Xenopus* oocytes show that the mutations all cause similar gain-of-function, namely that instead of being an ion pump, the mutations in *ATP1A1* make the Na,K-ATPases into ion channels that allow sodium or protons (depending on the specific mutation) to flow into the cell. Potassium is not (or for the TM9 deletions, only to a minor extend) transported, and physiological potassium levels have negligible effect (Azizan et al., 2013).

The effect of turning a pump into a channel is much more severe than simply inactivating the pump. The *ATP1A1* mutations clearly show this—only regions where mutations cause the pump to become a cation channel have been reported in APAs, while many of the disease-causing mutations in *ATP1A2* and *ATP1A3* seem to impair pump function (cf. below). Pharmacologically, the same is evident—pump inhibitors like cardiotonic steroids are toxic, but not nearly as toxic as a huge molecule produced in *Palythoa* corals, palytoxin, which binds the sodium pump and turns it into a channel by causing the inner and outer gates to open simultaneously. Palytoxin is the second deadliest non-peptide molecule known, since just a single molecule on a cell surface can dissipate the cell's ionic gradient (Rossini and Bigiani, 2011).

### Alpha2 in disease

**Figure 4:**
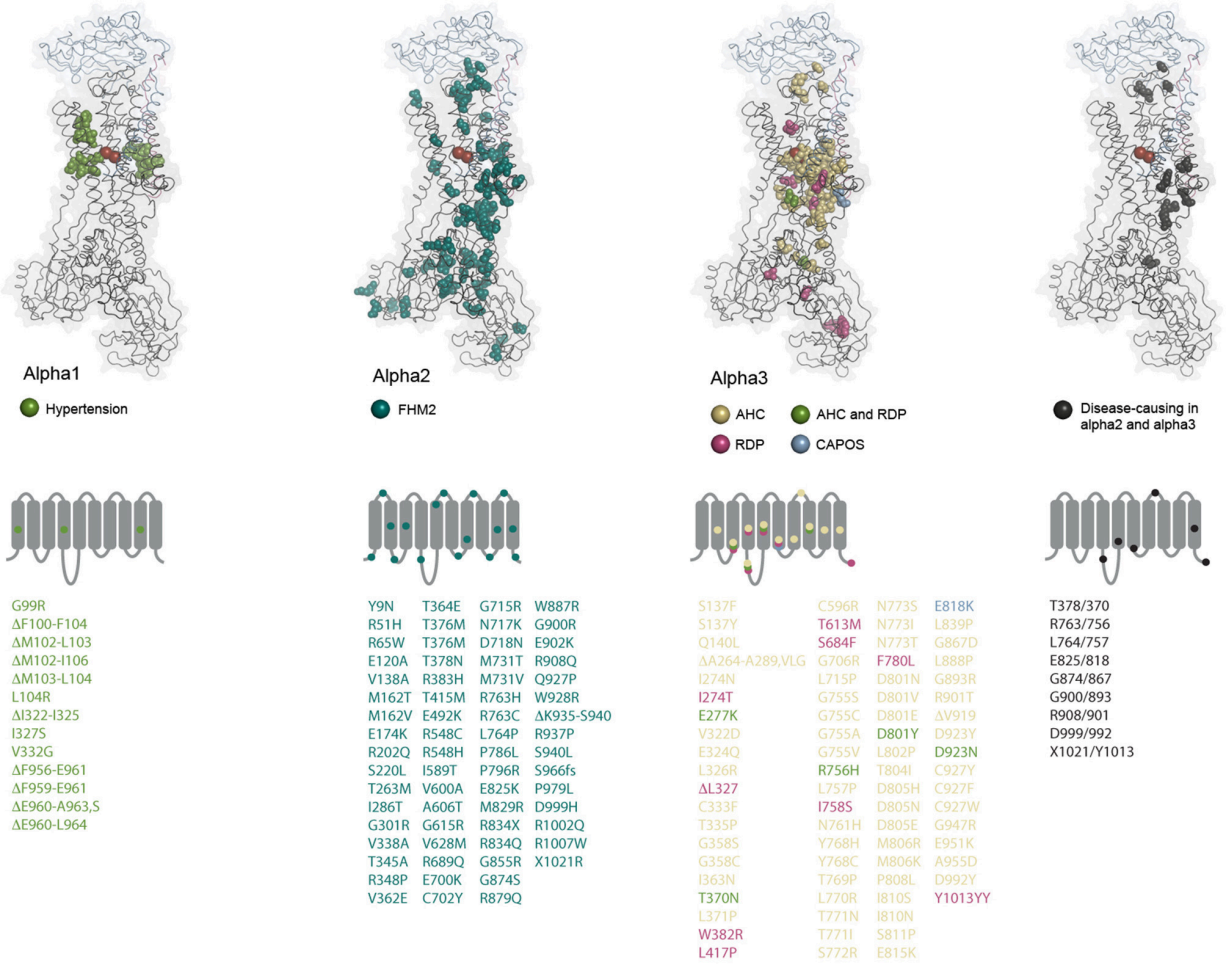
Structural maps of the disease-causing mutations. The amino acids altered by disease-causing mutations in the three alpha subunit genes are shown as spheres and color-coded as indicated for each subunit. *ATP1A2* and *ATP1A3* mutations that affect similar positions in the two alphas are also indicated. The alpha1 amino acid alterations have not been reported in the other alphas. The residues are mapped on the potassium-occluded pig kidney structure 2ZXE (Shinoda et al., 2009) (alpha in black cartoon, beta in blue cartoon, gamma in purple cartoon, potassium ions as red spheres). In alpha1, all deleted residues are indicated. In alpha2 and 3, only cases with deletion of single residues are indicated. Under each subunit, it is schematically indicated which loops and TM helices are targeted by mutations. Multiple mutations affecting residues in a single segment are indicated by one mark if they cause the same disease. The mutations are listed underneath using the same color coding, fs: frameshift.

Familial Hemiplegic Migraine (FHM) is an autosomally inherited form of migraine where the patients experience aura and weakness in one side of the body during attacks. FHM-causing mutations have been identified in three genes encoding a calcium channel (FHM1), a sodium channel (FHM3) and the alpha2 subunit (FHM2) (De Fusco et al., 2003). At least 80 mutations in *ATP1A2* have been described to cause FHM2 (Figure 4, reviewed in Bottger et al., 2012; Pelzer et al., 2016). In contrast to the *ATP1A1* mutations that all target recurrent hotspots, many of the mutations in *ATP1A2* have been reported just once, and they affect both the transmembrane and the cytoplasmic parts of the protein. Most of the mutations that have been characterized cause loss or reduction of function, either because the ATPase function of the pump is compromised or because ion binding and transport are affected, but many of the mutations also impair trafficking of the pump to the membrane (Morth et al., 2009, Spiller and Friedrich, 2014).

The Na,K-ATPase in astrocytes with alpha2 and the beta2 subunit has relatively low potassium affinity and is suggested to be particularly important when the extracellular potassium concentration is high (Larsen et al., 2014). In agreement with this suggestion, mouse models with FHM2 mutations knocked in show reduced clearance of potassium and glutamate (Bottger et al., 2016; Capuani et al., 2016). Elevated potassium and glutamate levels are known to augment cortical spreading depression, which is suggested to cause some of the symptoms experienced by patients having an attack of migraine with aura, and enhanced spreading depression was measured in the knock-in mice (Lauritzen et al., 2011). Cortical spreading depression is characterized by a wave of lack of neuronal activity that travels across the cerebral gray matter after a brief period of hyperexcitability. Normally, astrocytes protect against cortical spreading depression because they take up potassium and glutamate (Lauritzen et al., 2011), but when there is a high need for restoring of the ionic gradients and only one allele from which a fully functional alpha2 can be expressed, the astrocyte may not be able to fulfill that role to satisfaction: the Na,K-ATPase is both directly involved in potassium clearing and indirectly in glutamate clearing, because the glutamate transporter uses the energy from co-transport of three sodium ions and counter-transport of one potassium ion to transport one molecule of glutamate.

### Alpha3 in disease

Autosomal dominant mutations in *ATP1A3* were first shown to cause Rapid-onset Dystonia Parkinsonism (RDP, de Carvalho Aguiar et al., 2004), and later two other neurological syndromes, Alternating Hemiplegia of Childhood (AHC, Heinzen et al., 2012; Rosewich et al., 2012) and CAPOS (cerebellar ataxia, areflexia, pes cavus, optic atrophy, and sensorineural hearing loss, Demos et al., 2014). Although the three syndromes were originally identified as phenotypically distinct, it has become clear that many patients do not strictly fall into one category or the other, but may have symptoms that fall on a continuous spectrum as well as unique symptoms for individual mutations (Rosewich et al., 2014; Paciorkowski et al., 2015; Sweney et al., 2015; Kanemasa et al., 2016; Liu et al., 2016; Smedemark-Margulies et al., 2016; Sweadner et al., 2016).

RDP is characterized by a triggering, stressful event causing sudden (hours to days) onset of irreversible rostrocaudal gradient of dystonia with parkinsonism. The onset is typically caused by physical or emotional stress in early adulthood, and in addition to the motor effects, most patients have psychiatric symptoms (Brashear et al., 2012). The patients are unresponsive to L-DOPA, and there is no drug therapy available.

AHC patients are typically diagnosed before 18 months of age from recurring episodes of weakness or paralysis of one side of the body. The attacks can vary in length from minutes to days and are typically combined with additional paroxysmal symptoms like dystonia, choreoathetosis, nystagmus and about half of the patients have epileptic seizures. During sleep, symptoms disappear (Heinzen et al., 2014). AHC is further associated with progressive deterioration of the patient's health between attacks, including developmental delay, retardation, hypotonia and compromised motor skills. For treatment, the calcium channel blocker flunarizine may reduce the length and severity of attacks in some patients, and benzodiazipines may have positive effect, possibly because they induce sleep. A ketonic diet has also been reported to stall disease progression (Roubergue et al., 2015).

CAPOS syndrome typically starts in childhood when fever triggers the disease, which is characterized by cerebellar ataxia, areflexia, and progressive loss of sight and hearing. Nystagmus and hypotonia are also common symptoms (Heimer et al., 2015).

For all of the three diseases, it is clear that the *ATP1A3* mutations have high penetrance, but also that the genetic background of an affected individual is important for its manifestations. Because of the severity of RDP and AHC, the mutations are typically *de novo*, while CAPOS is also inherited.

There are now close to 80 different disease-associated mutations reported in *ATP1A3* (Figure 4). At some positions, amino acid changes cause RDP as well as AHC, but most of the mutations are unique for one of the diseases, and there are genotype-phenotype correlations: all CAPOS patients sequenced have the same, single mutation, E818K, and all people identified with the mutation have CAPOS. For AHC and RDP, *ATP1A3* mutations have been found in most but not all of the sequenced patients, so it remains to be determined if mutations in other genes can give similar symptoms. Strikingly, just two mutations, D801N and E815K, account for up to two thirds of the AHC patients, while the RDP-causing mutations seem to target the protein broadly with no obvious hotspots (Figure 4). Compared to the disease-causing mutations in *ATP1A1* and *ATP1A2*, RDP is in that respect more similar to FHM2 where many of the mutations may be associated with loss-of-function, while few hotspot positions give the phenotypes in AHC and CAPOS, as also seen for hypertension-causing *ATP1A1* mutations. It remains to be determined whether alpha3 gains any novel functions in AHC or CAPOS patients, but it has been suggested that the AHC-causing mutations may exert a dominant negative effect on the alpha3 expressed from the healthy allele by an unknown mechanism (Li et al., 2015).

Markedly, none of the mutations that change alpha1 from a pump to a channel have been described for the other genes, probably because that would be too severe an alteration. A few of the mutations in *ATP1A2* and *ATP1A3* target the same positions (Figure 4), but it is clear from the structural mapping that the *ATP1A3* mutations often target the ion binding sites, while that is not the case for *ATP1A2*, possibly suggesting that impaired ion binding in alpha2 would be incompatible with life or that they cause other symptoms than FHM.

## Conclusion

The Na,K-ATPase was first described 60 years ago by Jens Christian Skou (Skou, 1957), but novel insight into the pump's atomic structure, cellular regulation and pathophysiological roles continues to emerge, and many aspects await future studies. The various subunit isoforms are optimized for the specific requirements and challenges that different cell types face for maintaining ionic homeostasis: the subunit variation allows for fine-tuning of the basic kinetic properties of the pump as elucidated in many studies, but much remains to be learned about how the variation determines interactions with other cellular partners and thereby regulates e.g., the activity, localization and stability of the pump. Furthermore, the amazingly improved possibilities for sequencing patient DNA will likely disclose novel links between mutations affecting alpha subunits and pathophysiological conditions: Adenomas other than the adrenal may gain advantage from somatic mutations affecting the alpha1 subunit, and mutations altering e.g., the ion binding residues in alpha2 may confer diseases other than FHM2. It is also still enigmatic why the different mutations in the alpha3 gene cause such varied diseases, and since alpha4 is essential for sperm function, mutations in its gene are highly likely to affect the male bearers' fertility. It further remains to be determined whether mutations in the beta or FXYD subunit genes are associated with diseases.

## Author contributions

MC, FH, and HP discussed, wrote and edited the manuscript and made the tables and figures.

### Conflict of interest statement

The authors declare that the research was conducted in the absence of any commercial or financial relationships that could be construed as a potential conflict of interest. The reviewer PMV and handling Editor declared their shared affiliation, and the handling Editor states that the process nevertheless met the standards of a fair and objective review.
